# 4-D single particle tracking of synthetic and proteinaceous microspheres reveals preferential movement of nuclear particles along chromatin – poor tracks

**DOI:** 10.1186/1471-2121-5-45

**Published:** 2004-11-23

**Authors:** Christian P Bacher, Michaela Reichenzeller, Chaitanya Athale, Harald Herrmann, Roland Eils

**Affiliations:** 1 Division of Theoretical Bioinformatics, Deutsches Krebsforschungszentrum, Im Neuenheimer Feld 580, 69120 Heidelberg, Germany; 2 Division of Cell Biology, Deutsches Krebsforschungszentrum, Im Neuenheimer Feld 580, 69120 Heidelberg, Germany; 3 Complex Biosystems Modeling Laboratory, Massachusetts General Hospital, Harvard Medical School, Charlestown MA 02129, USA

## Abstract

**Background:**

The dynamics of nuclear organization, nuclear bodies and RNPs in particular has been the focus of many studies. To understand their function, knowledge of their spatial nuclear position and temporal translocation is essential. Typically, such studies generate a wealth of data that require novel methods in image analysis and computational tools to quantitatively track particle movement on the background of moving cells and shape changing nuclei.

**Results:**

We developed a novel 4-D image processing platform (TIKAL) for the work with laser scanning and wide field microscopes. TIKAL provides a registration software for correcting global movements and local deformations of cells as well as 2-D and 3-D tracking software. With this new tool, we studied the dynamics of two different types of nuclear particles, namely nuclear bodies made from GFP-NLS-vimentin and microinjected 0.1 μm – wide polystyrene beads, by live cell time-lapse microscopy combined with single particle tracking and mobility analysis. We now provide a tool for the automatic 3-D analysis of particle movement in parallel with the acquisition of chromatin density data.

**Conclusions:**

Kinetic analysis revealed 4 modes of movement: confined obstructed, normal diffusion and directed motion. Particle tracking on the background of stained chromatin revealed that particle movement is directly related to local reorganization of chromatin. Further a direct comparison of particle movement in the nucleoplasm and the cytoplasm exhibited an entirely different kinetic behaviour of vimentin particles in both compartments.

The kinetics of nuclear particles were slightly affected by depletion of ATP and significantly disturbed by disruption of actin and microtubule networks. Moreover, the hydration state of the nucleus had a strong impact on the mobility of nuclear bodies since both normal diffusion and directed motion were entirely abolished when cells were challenged with 0.6 M sorbitol. This effect correlated with the compaction of chromatin. We conclude that alteration in chromatin density directly influences the mobility of protein assemblies within the nucleus.

## Background

Interphase nuclei are structurally highly organized with chromosomes restricted to defined territories[1]. The movement of large complexes or nuclear bodies such as Cajal bodies or PML bodies in the nucleus has been described by various groups [2-4]. This type of organization of interphase chromosomes indicates that the resulting interchromatin compartment provides a so-called interchromosomal domain (ICD) space that differs significantly from that occupied by chromatin [5]. It was shown that nuclear bodies as well as specific RNA are excluded from the chromosome territories but reside in an interchromatin compartment [5-7]. Investigation of the diffusional accessibility of the nucleus for microinjected DNA and dextrans of varying molecular weight by fluorescent recovery after photobleaching (FRAP) methods revealed significant differences in mobility according to size. FITC-dextrans of molecular sizes up to 580 kDa were demonstrated to be fully mobile, whereas DNA fragments were nearly immobile [8]. Furthermore, a size- and electrical charge-dependent exclusion for macromolecules is encountered for chromatin regions [9]. In contrast, poly(A) RNA has been shown to move freely throughout the interchromatin space of the nucleus with properties characteristic of diffusion [10]. Moreover, the large ribosomal subunits seem to exhibit a random movement in a Gaussian manner without evidence for any direct path on their way from the nucleolus to the nuclear pores [11]. Recently, microinjection of fluorescent nanospheres has been used to track the movement of such particles under distinct experimental conditions [12]. The authors employed a silicon – intensifier target camera coupled to an epifluorescence microscope in combination with a 2-D particle – nanotracking routine implemented in the Metamorph / Metaview image processing system [13,14]. In particular, tracking of nanospheres within the nucleus revealed that the particles diffused freely in restricted "cages", eventually translocating into another "cage". These studies, however, did not reveal any information on the 3-D movement of particles in comparison with local chromatin density. Such a study requires recording of simultaneous time-lapse recording of three-dimensional image stacks of particles and chromatin using a confocal laser scanning microscope as described in the present study.

Kinetic analysis of nuclear bodies requires spatio-temporal microscopic imaging of live cells generating a huge amount of data that is only difficult or impossible to analyze in a standardized way without computational support. The present developments of an Open Microscopy Environment (OME) aims at providing a standardized informatics solution for the storage, management and analysis of light microscopic image data [15]. For quantitative analysis of complex data from live cell experiments a variety of systems have been developed (for review see [16]). An integrated image analysis solution should include tools for all steps in the image processing chain, i.e. image preprocessing and segmentation of objects, registration of moving and deforming cells, tracking of objects over time, and multi-dimensional visualization and kinetic analyzes of dynamic objects. Only with the availability of quantitative kinetic data it is possible to obtain insight into the underlying mechanisms of biological processes such as those involved in the functional and spatial organization of the cell nucleus.

In the present study we describe a combined computational and experimental approach to study the dynamic behaviour of nuclear body-like particles formed by GFP-NLS-vimentin [17] in response to different cellular inhibitors and, most importantly, in relation to the chromatin structure of the nucleus. This has been compared with the motion of polystyrene particles of similar size. Since both kinds of "bodies" display identical movement, the vimentin bodies can be regarded and hence employed as entities supposedly "biologically inert" for the nucleus. Using our novel image processing platform TIKAL we show that the kinetics of nuclear particles are influenced by various cellular inhibitors. Furthermore we show that the kinetics of nuclear bodies is directly influenced by local restructuring of chromatin domains.

## Results

### An experimental system for probing particle kinetics in the cell

**Figure 1:**
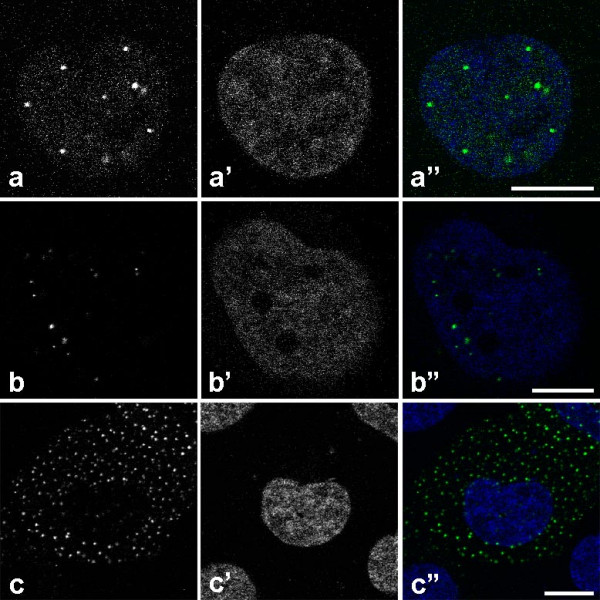
A) Nucleus of a SW13 cell stably transfected with an expression plasmid encoding for GFP-NLS-vimentin. B) SW13 cell nucleus containing microinjected 100 nm-microspheres. C) SW13 cell transfected with GFP-vimentin showing cytoplasmic particle formation. A', B', C'): Corresponding Hoechst 33342 chromatin stain, A", B", C") superimposed images.

We used fast 3-D time-lapse confocal laser scanning microscopy to analyze the mobility of *Xenopus laevis *GFP-NLS-vimentin and synthetic particles (polystyrene microspheres) within the nucleoplasm. GFP-NLS-vimentin is deposited at 37°C within the nucleus of stably transfected SW13 cells in multiple discrete bodies (8 – 40). On average the cells contain bodies that are nearly 1 μm in diameter as observed in the light microscope (Figure 1a). This corresponds to a particle diameter of about 200 – 500 nm in fixed cells as prepared for conventional electron microscopy (data not shown). From correlative light and electron microscopy studies we know that nuclear vimentin particles are excluded from dense chromatin regions (Richter et al., submitted). In contrast, transfection of human vimentin-free SW13 cells with an expression plasmid encoding GFP-vimentin without the engineered NLS sequence leads to the formation of many cytoplasmic particles (> 100) of very similar size (Figure 1c).

To study the nucleoplasmic mobility of particles of a defined size we microinjected unloaded polystyrene beads into the nucleus of living cells. We used orange fluorescent 100 nm-beads that resemble in size authentic nuclear bodies such as PML- or Cajal bodies [3,18-20] on the light microscopic level. Thereby we attempted to find out how the mobility of the ectopically expressed nuclear vimentin particles related to polystyrene particles [17,21].

In the course of these studies we found that a system based on an ectopically expressed protein has several clear advantages compared to the microinjection of beads. First, the expression efficiency of the GFP-vimentin construct is very high. More than 50% of the cell nuclei show formation of nuclear vimentin bodies. Since the cells are stably transfected they reflect a normal physiological state. In contrast, for microinjection only approximately 10% – 20% of injected the cells survived over night culture (n = 300). Additionally, the artificial microspheres have to undergo tedious processing steps such as sonification and centrifugation prior to injection to avoid the formation of aggregates.

### A computational system for tracking nuclear particles on the background of moving cells

For the analysis of complex data derived from spatio-temporal imaging of trafficking particles we developed a proprietary image processing platform, TIKAL (see Methods). The platform allows to directly and easily handle complex microscopic data and to dynamically interact with the data set throughout the whole quantitative data analysis steps. The image processing pipeline is initiated by image pre-processing steps including noise reduction followed by object segmentation (for details see Methods).

In many cases, cells move and also change their morphology during the observation period. Global movements include translocation and rotation, whereas morphological changes are either caused by global changes in size (affine transformation) or by local deformations. Since any of these transformations overlay the actual movement of nuclear particles within the cell, we corrected for them by rigid transformations (translocation and rotation), affine transformations (scaling) and by thin-plate spline models (local deformations; [22,23]; for details see Methods). These transformations allow a direct measurement of nuclear particle movements without any bias induced by external forces and cellular movements.

**Figure 2:**
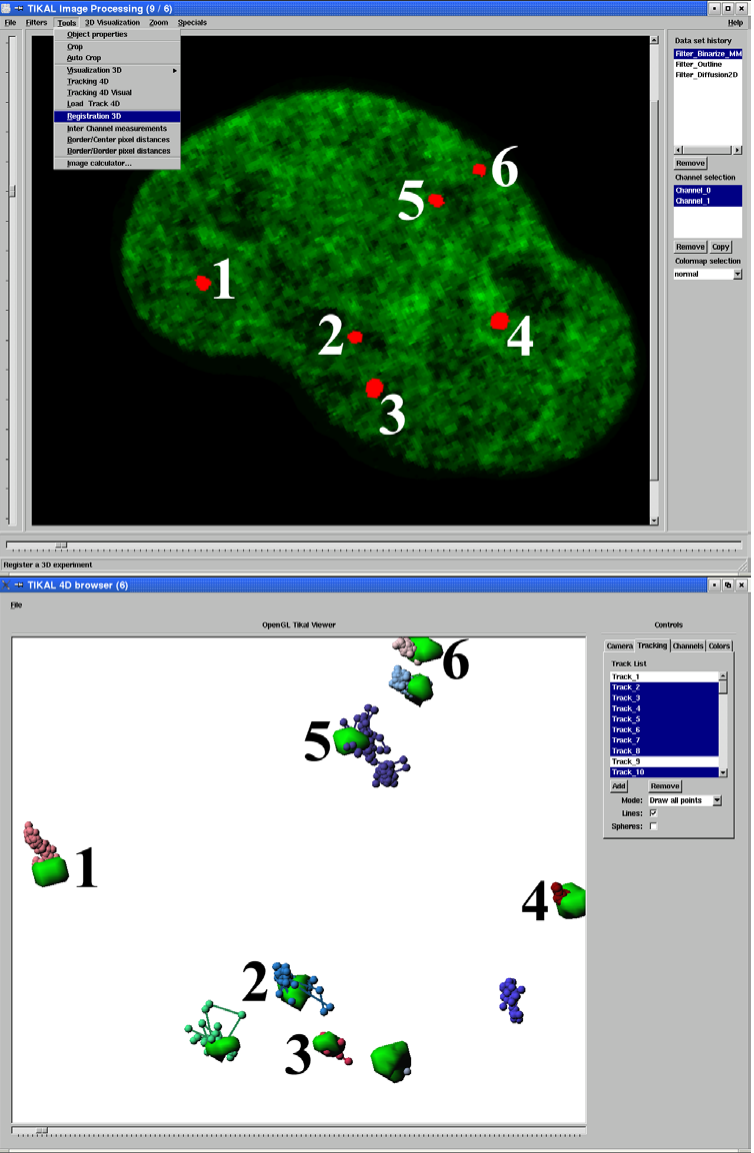
Screen shot of the image processing platform TIKAL. (top) Image shows a sample two-dimensional section through a nucleus with binarized nuclear particles (red) counterstained with Hoechst 33342 stain (green). Pull down menu exemplifies different tools for quantitative analysis integrated into TIKAL. Numbers indicate different nuclear particles reconstructed by 3D isosurface reconstruction (bottom). Computed tracks of nuclear bodies over time are displayed as spheres on a string in the multi-dimensional scene viewer.

For quantitative evaluation of kinetics of moving particles we extended our single particle tracking approach formerly developed for two-dimensional time series [24] to automatically track objects in 3-D time series. The automatically computed 4-D tracks are visualized together with a surface rendered 3-D reconstruction of segmented nuclear particles in a multi-dimensional scene viewer (Figure 2). By interacting with the automatically computed trajectories the user is able to interactively control and correct for possible artefacts during the tracking procedure, e.g. deriving from noisy images. Applying TIKAL, we rapidly reconstructed, visualized and analyzed the trajectories of 1131 particles in more than 50 cells.

### In vivo observation of microspheres

**Figure 3:**
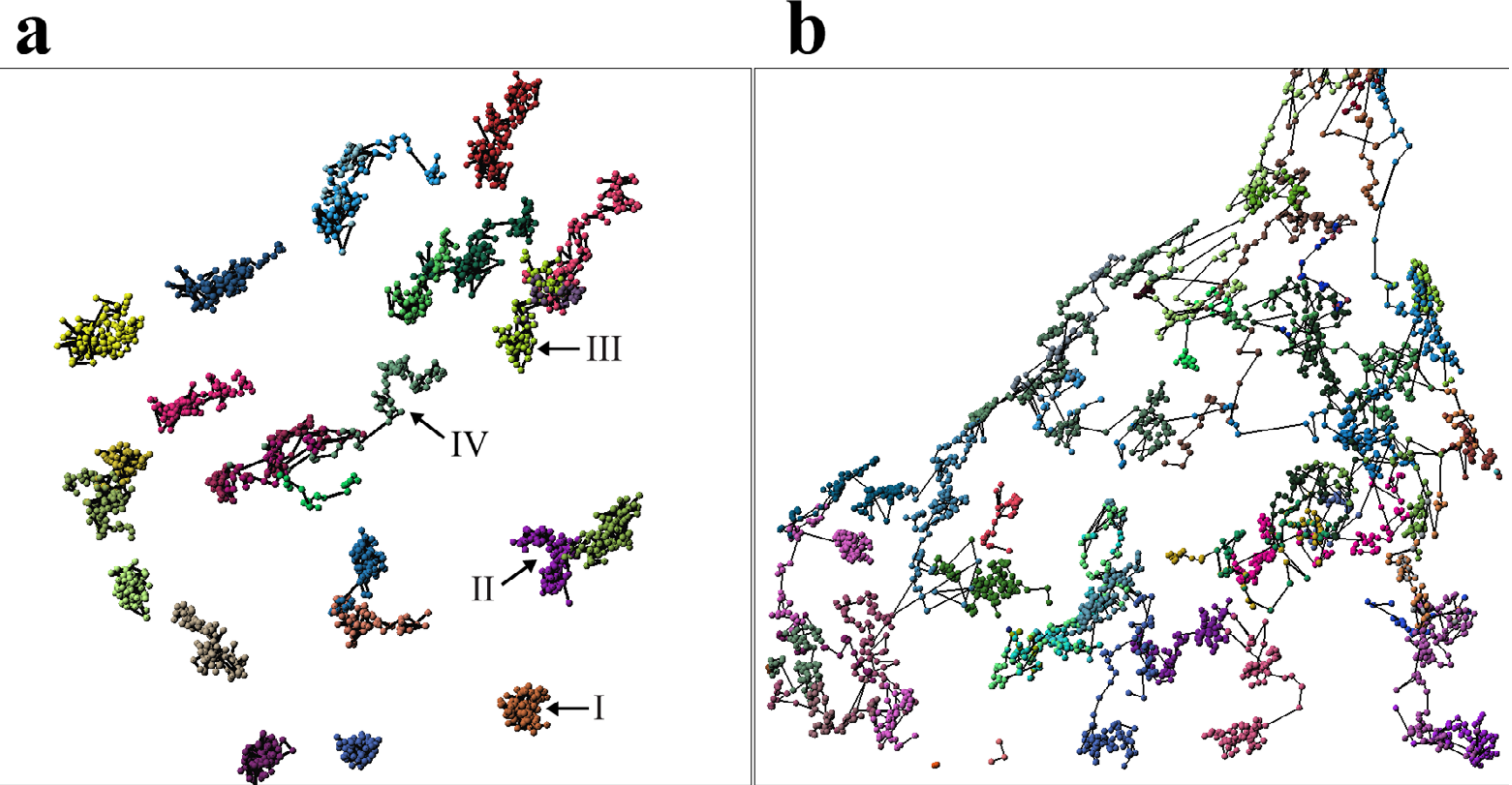
4-D visualization of vimentin particle trajectories a) in the nucleus and b) the cytoplasm. Each color represents an individual vimentin body. The respective centers of masses are indicated as spheres. Tracks are symbolized by interconnecting lines. Arrows indicate the different kinds of diffusion (see Figure 4a). Major types of movement are indicated: I) confined diffusion; II) obstructed diffusion; III) normal diffusion; IV) directed motion.

**Figure 4:**
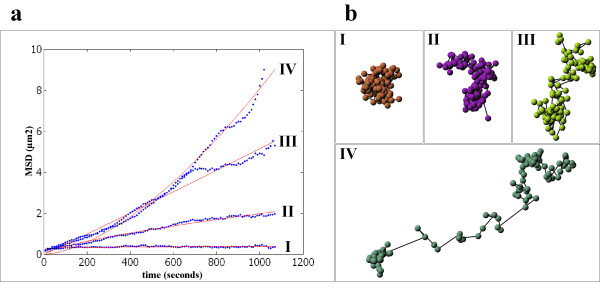
Different classes of mean square displacement of tracked nuclear vimentin particles. a) Mean squared displacement (MSD) of four representative modes of particle mobility (x-axis: acquisition time in seconds; y-axis: MSD). Roman numbers: I) confined diffusion; II) obstructed diffusion; III) normal diffusion; IV) directed motion (The numeration refers to indicated trajectories in Figure 3a). b) The four different mobility classes are represented by particle trajectories. The trajectories correlate to the numbers indicated by arrows in Figure 3a.

**Figure 5:**
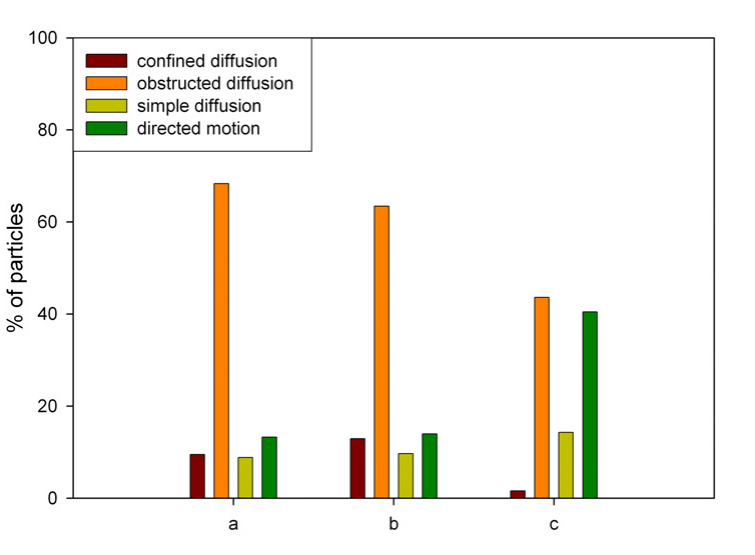
Classification of particles into four groups of diffusional motion according to cellular localization and their calculated anomalous diffusion coefficient. a) nuclear vimentin particles, (b) microinjected nuclear 100 nm microspheres, (c) cytoplasmic vimentin particles.

We imaged the microinjected microspheres and the GFP-NLS-vimentin particles in SW13 cells over a time interval of 20 min (Figure 1a and 1b). After image processing a qualitative analysis of the trajectories of 154 microspheres visualized in 12 cells suggested the same kind of mobility for both the 100 nm-beads and the GFP-NLS-vimentin bodies (Figure 3a). For a more rigorous quantitative comparison the mean square displacement (MSD) was calculated for each individual particle as well as its anomalous diffusion coefficient α. Based on α, the analyzed particles were classified into four arbitrary groups of mobility using the theoretical framework from previous studies [25,26]: (i) confined diffusion (α < 0.1), (ii) obstructed diffusion (0.1 ≤ α < 0.9), (iii) simple diffusion (0.9 ≤ α < 1.1) and (iv) directed motion (α ≥ 1.1) (for sample trajectories see Figure 4). The comparison of the calculated anomalous diffusion coefficients of the GFP-NLS-vimentin bodies with those of the 100 nm-microspheres revealed no significant changes in the distribution (p = 0.126; compare Figure 5a and 5b. For statistical significance of interexperimental differences of distribution patterns for anomalous diffusion coefficients refer to Table 1).

**Table 1:** Significance analysis of interexperimental differences in distribution of anomalous diffusion coefficients by Kolmogorov – Smirnov test Statistical significance analysis for interexperimental differences in cumulative distribution of anomalous diffusion coefficients (for corresponding distribution plots see Figure 5 and Figure 9) was performed by the Kolmogorov – Smirnov test [45]. The different experiments are depicted as followed: control = vimentin particles in the nucleus; beads = sepharose microspheres (100 nm) in the nucleus; cytoplasm = vimentin particles in the cytoplasm; Vimentin particles in the nucleus with drug treatment: azide / deoxyglucose (10 mM / 50 mM); cytochalasin D (1 μg/ml); nocodazole (0.04 μg/ml); sorbitol (600 mM).

Experiment 1	Experiment 2	p-value
control	beads	0.1264
control	cytoplasm	1.11E-15
beads	cytoplasm	5.22E-08
control	azide / deoxyglucose	
control	cytochalasin D	0.01643
control	nocodazole	9.70E-05
control	sorbitol	1.78E-14
azide / deoxyglucose	cytochalasin D	0.1095
azide / deoxyglucose	nocodazole	0.004754
azide / deoxyglucose	sorbitol	8.45E-11
cytochalasin D	nocodazole	0.7306
cytochalasin D	sorbitol	2.00E-09
nocodazole	sorbitol	7.96E-09

Next, we were interested in the differences of the kinetic behaviour of the *Xenopus *GFP-vimentin particles in the nucleoplasm as compared to that in the cytoplasm. Transfected SW13 cells lack endogenous vimentin and therefore do not have intermediate filaments. Instead, only small spherical aggregates of the temperature-sensitive amphibian protein were deposited throughout the cytoplasm (Figure 1c; [27,28]). When directly comparing the particle trajectories of the nucleoplasmic vimentin bodies to the cytoplasmic vimentin bodies striking differences were found. Nuclear-targeted vimentin particles displayed a spatially restricted movement within distinct corrals. However, on occasion they were able to move spontaneously to an adjacent corral (Figure 3a). The maximum distance that a NLS-vimentin particle moved was 4 μm within the observation time of 20 min. This corresponds on average to a speed of 0.2 μm / min. Most strikingly, we never encountered crossing nuclear trajectories. In contrast, cytoplasmic vimentin particles moved along more extended trajectories and did hardly ever exhibit corralling events (Figure 3b). Moreover, the cytoplasmic bodies moved three times as fast, i.e. up to 12 μm in distance within 20 minutes.

A comparison of the overall kinetic characteristics of nuclear vimentin bodies versus sepharose beads revealed that in the nucleus obstructed diffusion is the major type of movement whereas in the cytoplasm directed motion is observed to a similar extent, both accounting for approximately 40 %. Notably, confined diffusion is very rarely found in the cytoplasm whereas in the nucleus 11.4 % of the movement can be accounted for it (Figure 5, Table 1).

### Chromatin remodelling directly effects mobility of nuclear particles

**Figure 6:**
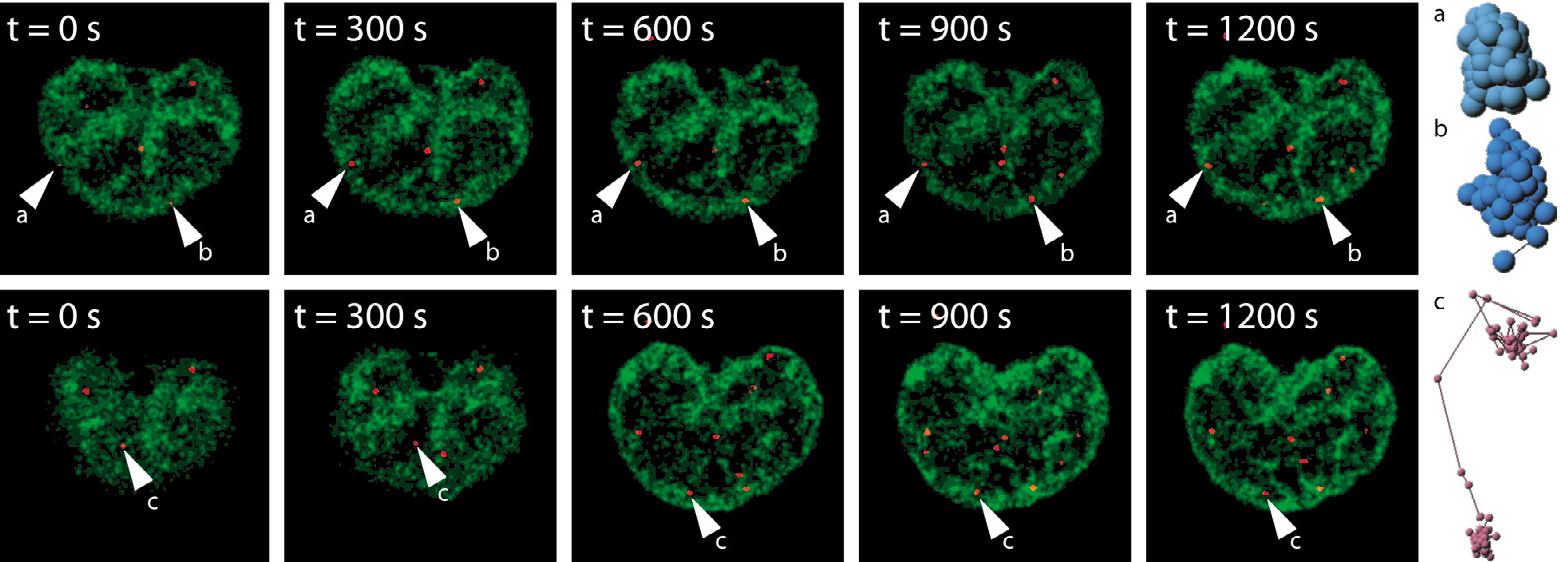
4-D – tracking of vimentin particles in the nucleus. (a, b) particles with restricted movement (confined diffusion) c) corralled particle and corresponding trajectories. (red = vimentin bodies; green = Hoechst 33342 staining).

**Figure 7:**
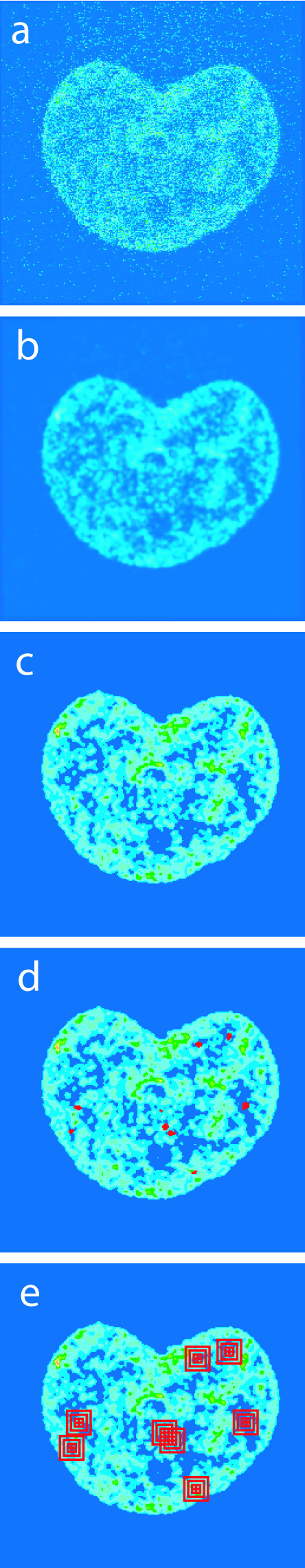
Chromatin intensity analysis showing the preprocessing steps for the Hoechst 33342 images: a) unprocessed original image; b) Gaussian smoothing; c) image classified into 8 regions of grey values; d) localization of particles in the cell nucleus (red); e) measuring the mean grey value intensity around the center of mass for each individual vimentin particle (see Methods for a complete description).

**Figure 8:**
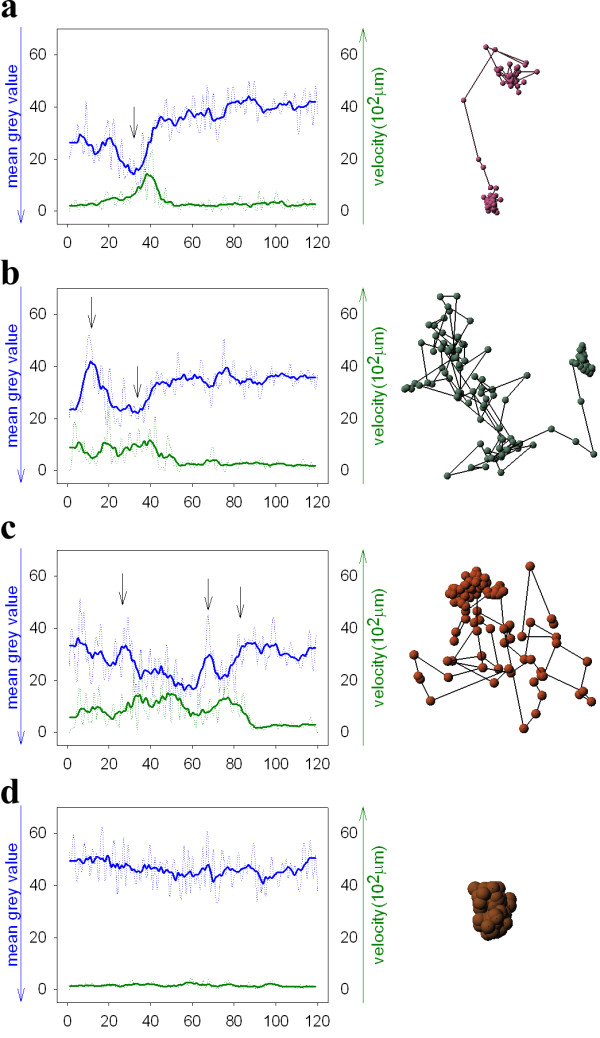
Correlation plots between mean grey values (green line) of chromatin densities and particle velocity (blue line) over a time range of 20 minutes. Arrows indicate significant changes in intensity and particle velocity. Red: NLS-GFP-vimentin; green: Hoechst 33342 stain.

In the next step we analyzed the influence of chromatin density on mobility of nuclear particles. Upon inspection of corralled versus highly mobile nuclear particles (Figure 6) we frequently observed a correlation between chromatin density in the neighbourhood of particles and their degree of motility. For a more rigorous quantitative analysis the mean grey value in a neighbourhood of 9 × 9 pixels was measured for each particle over an observation time of 20 min with a time-lapse of Δt = 10 seconds (Figure 7). Evidently, there is a strong correlation between chromatin density and particle velocity. Particles with high velocities were exclusively formed in areas of very low chromatin density. An increase in chromatin density directly led to a decrease of particle velocity (Figure 8a and 8b). A similar reverse effect was detected in cases where particles had very low velocities. After release of a body from a dense chromatin cluster a sudden increase of its mobility could be observed. In this case a high chromatin density was measured during the resting phase of the body, whereas a decrease of chromatin density was detected before the particle started to increase its velocity (Figure 8c). This phenomenon was prominently encountered with particles showing a high frequency of changes in corralled and more directed movement (Figure 8a,8b,8c). For particles with minimal changes in distance and velocities, a constant chromatin density with low fluctuation in measured grey values was observed (Figure 8d).

### Influence of inhibitors on the mobility of nuclear vimentin bodies

In order to investigate the contribution of structural elements of the cytoplasm to nuclear body mobility in living cells, inhibitors of cellular energy as well as drugs that lead to the depolymerization of cytoskeletal systems were employed. In particular, we inhibited cellular ATP production and incubated cells with agents that depolymerize microtubules or microfilaments, both of which are tracks for molecular motors in the cytoplasm. Cells were imaged prior to addition of the inhibiting substance for 10 minutes with image stacks acquired every Δt = 10 seconds.

**Figure 9:**
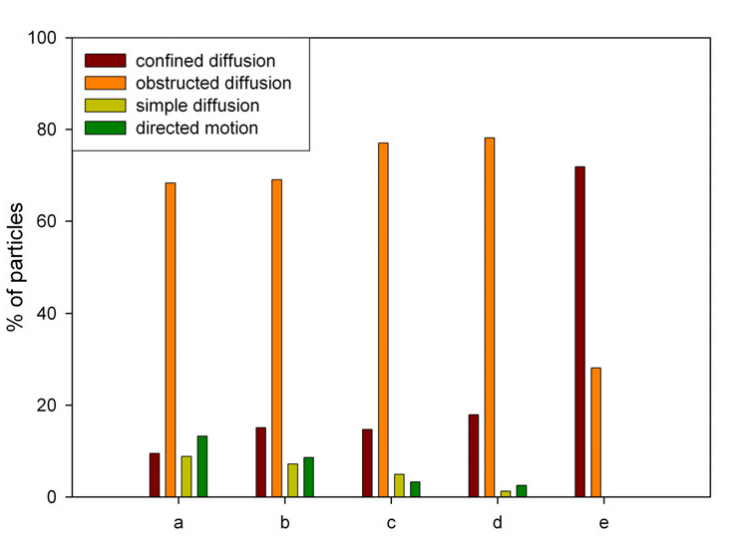
Mobility of nuclear vimentin bodies under the influence of inhibitors. Classification was performed according to the diffusion coefficient. a) control; b) azide / deoxyglucose (10 mM / 50 mM); c) cytochalasin D (1 μg/ml); d) nocodazole (0.04 μg/ml); c) sorbitol (600 mM).

In a first step, the dependency of nuclear vimentin particles on energy-dependent mechanisms as investigated by depletion of ATP through incubation with 10 mM azide and 50 mM deoxyglucose followed by live cell imaging over a time interval of another 10 minutes. For more than 140 bodies in eight cells the diffusion coefficients were calculated. Compared to the control group (Figure 5a, 9a and Table 1) an absolute increase of 5.7 % for confined diffusion, an absolute increase of 0.6 % for obstructed diffusion, an absolute decrease of 1.7 % for simple diffusion and an absolute decrease of 4.6 % for directed motion were observed (Figure 9b, Table 1). Interestingly, after addition of azide / deoxyglucose a rapid condensation of chromatin was observed. Chromatin condensation was reversed after removal of the inhibitor as also reported recently [29], [46].

Secondly, the impact of the presence of a functional actin cytoskeleton on vimentin body movement was tested using the actin polymerization inhibitor cytochalasin D at 1 μg/ml (Figure 9c, Table 1). Five cells were imaged after addition of cytochalasin D every Δt = 10 seconds for 10 minutes. A quantitative analysis of 121 bodies revealed an absolute increase of 5.3 % and 8.6 % for confined and obstructed diffusion, respectively, and an absolute decrease of 3.9 % for simple diffusion and a decrease of 10.0 % for directed motion compared to the control group.

To study the role for microtubule structures on particle mobility we used the microtubule polymerization inhibitor nocodazole (Figure 9d, Table 1). The effect of nocodazole treatment (0.04 μg/ml) was also imaged in 5 cells with image stacks every Δ*t *= 10 seconds for 10 minutes. By analysing 119 bodies, an absolute increase of 8.7 % and 9.9 % was detected for confined and obstructed diffusion while an absolute decrease of 7.7 % and 10.9 % compared to the control groups was observed for simple diffusion and directed motion, respectively.

### Particle movement in dehydrated cells

Finally the dependency of the GFP-NLS-vimentin mobility on availability of water in the nucleus was tested by treating the cells with sorbitol (600 mM) [30] (Figure 9e, Table 1). In contrast to the previous inhibitors, we observed a dramatic change in particle mobility. Calculation of diffusion coefficients for 101 particles in five cells revealed the total loss of simple diffusion and directed motion activities. Accordingly, 79.1 % of all particles were found in the confined diffusion and 20.9 % in the obstructed diffusion group.

In summary kinetic changes were most prominent for the directed motion mode. ATP depletion decreased directed motion about 30 % relative to the control group. Treatments with cytochalasin D and nocodazole even showed a 70 % decrease in directed motion relative to the control group. The most striking effect was encountered by treating the cells with sorbitol. Simple diffusion and directed motion were totally abolished whereas the number of particles exhibiting confined diffusion increased by a factor of 7 relative to the control group.

## Discussion

In this study we developed comprehensive bioinformatics tools to analyze the kinetic behaviour of small particles in the cell nucleus. For this purpose, fast time-lapse confocal laser scanning microscopy was used to record fluorescent particles in their chromatin environment. Automated image processing algorithms such as image registration and single particle tracking were instrumental to analyze the resulting complex data sets in a most efficient way. Usually, sophisticated image processing methods are widely not accessible for cell biology laboratories working with multi-dimensional data sets. A qualitative, interactive analysis of complex processes in living cells can yield interesting results. However, a quantitative insight into the underlying mechanisms can only be achieved by a rigorous computational analysis. While computational systems have been provided for estimating diffusion and binding constants based on photobleaching experiments of populations of small proteins [23,31-33], integrated software packages for single particle tracking of nuclear bodies on the background of moving and shape changing objects have not been provided yet to the community. Here, our system TIKAL closes an important gap. For an automated analysis of even larger sets of spatio-temporal data as in this study any software system needs to be adapted to data storage systems that are devoted for handling such huge data sets [15]. At the same time image analysis workflows have to be deployed onto computing clusters or the GRID ([34], accepted). Both developments are underway in our laboratory.

We visualized the different kinetic behaviours of nucleus-injected 100 nm polystyrene microspheres. Furthermore, a stably transfected cell line expressing GFP-NLS-vimentin, which forms nuclear particles in the same size range as microspheres, was used. The majority of nuclear particles moved with obstructed diffusion within distinct corralled regions. This kind of movement was essentially found also for microinjected polystyrene beads. The obstructed diffusion behaviour supports the notion that these particles can diffuse within corrals restricted by dense chromatin regions. Upon chromatin remodelling distinct less dense chromatin regions are formed and enable the particle to move to an adjacent corralled region. We were able to quantitatively assess this phenomenon by measuring the chromatin intensity around an individual particle. Our data show that chromatin intensity decreases prior to a global velocity increase of the particle. Therefore we conclude that the particles do not actively push their way through the chromatin. Moreover, the chromatin itself is able to support or induce the movement of individual particles. The ability of the particle to move from one corral to the next is restricted and regulated by the surrounding chromatin remodelling activities. However, whether local chromatin regions can actively influence the destination of small nuclear particle movement has to be resolved in future investigations. With the present assay we cannot discern whether changes in the velocity of a body simply correlate with the entry of a body into a domain or whether the changes are caused by interaction between a body and the surrounding chromatin domain surfaces.

The addition of cellular inhibitors caused significant changes in the diffusional behaviour of nuclear particles. In all treatments a reduction of active transport processes were observed. This suggests that the coordination of nuclear processes such as chromatin remodelling is not solely dependent on single factors such as ATP, i.e. ATP consuming enzymes. Moreover, chromatin regions in interphase nuclei apparently move in a diffusional way [19], while other factors such as cytoplasmic microtubules and actin filaments attached to the nuclear periphery possibly account for large-scale spatial chromatin rearrangements [35].

The phenomenon of energy dependent nuclear body movement has been also described in other studies where an anomalous diffusion behaviour and an ATP- and transcription-dependent association of Cajal bodies with chromatin was reported [2]. Further, upon ATP depletion in BHK cells rapid and large-scale movement of PML bodies stopped, whereas small localized movements of PML bodies were still observed [3]. A recent examination of the dynamic behaviour of PML nuclear bodies showed their fission to microstructures after different physiological stresses, and their fusion upon recovery [36]. Moreover it has been shown that movements of PML and other nuclear bodies can be described by diffusion of the individual body within a chromatin corral and its translocation resulting from chromatin diffusion [46]. However, future systematic studies will help to reveal the influence of drug treatments and cellular inhibitors on the dynamic behaviour of those and other nuclear bodies.

A further interesting observation was the reversible formation of chromatin dense regions upon energy depletion. The general effect of this reorganization seems to influence the mobility distribution pattern of the particles only slightly. However, we could observe a significant decrease in directed motion of up to 30 % upon inhibition of energy-depended processes.

Furthermore, in order to evaluate the degree of nuclear particle movement with respect to cytoplasmic dynamics, we used the vimentin system to analyze cytoplasmic particle mobility. Mostly active transport processes were observed. Two possible explanations for this phenomenon are suitable. Vimentin, which belongs to the group of intermediate filaments, forms crossbridges to other cellular structures. Since the SW13 cells lack endogenous expression of intermediate filament proteins such as cytokeratins and vimentin, possible interactions with these cytoplasmic intermediate filaments can be omitted. Specific interactions with dynein have been described [37,38]. Hence, newly synthesized vimentin is subjected to active transport processes and "guided" to cellular locations for the establishment of vimentin networks. Another explanation could assume that the filaments do not bind any cytoplasmic structure. In this less likely scenario the active transport of vimentin particles would result from the densely packed cytoplasm and the resulting pushing and pulling of adjacent actively transported molecules.

From our data we conclude that the NLS-vimentin system is very suitable for further studies of nuclear architecture. Though we obtained the same results with microinjection assays, the GFP-NLS-vimentin system has significant advantages such as the higher expression efficiency and the fact that the experiments can be performed in a cell system with normal proliferation characteristics.

## Conclusions

We presented a novel image analysis platform TIKAL that for the first time allows the 4-D tracking of nuclear particles on the background of moving and shape changing objects. TIKAL is complementary to other software systems designed for diffusion studies based on photobleaching experiments. Applying TIKAL we were able to analyze the dynamics of nuclear bodies under various different conditions and thus demonstrated that local chromatin remodelling accounts to a large extent for changes in the dynamics of individual nuclear bodies.

## Methods

### Expression plasmids and construction

The cloning of the *Xenopus laevi *GFP-NLS-vimentin expression plasmid has been described previously [17]. For the generation of the N-terminally tagged GFP-vimentin construct the vimentin cDNA was modified at the 5' – end to contain a BspE I – site followed by a Nde I – site containing the start methionine in frame subcloned into pBlueScript (Stratagene). The BspE I / BspE I fragment was then subcloned into pEGFP – C_1 _and the orientation verified by DNA sequencing.

### Cell culture and transfection

SW13 lacking endogenous vimentin [27] were usually grown in DMEM (Invitrogen, Karlsruhe, Germany) supplemented with 10% fetal calf serum (Seromed, Berlin, Germany), 20 mM glutamine and 100 μg/ml penicillin/streptomycin (Invitrogen) at 37°C and 5% CO_2_. For live cell imaging purposes the cells were resuspended in complete DMEM without Phenol Red (Invitrogen) and grown in 2- or 4-well Lab-Tek^® ^II chambers (Nalge Nunc International, Rochester, USA). Transient transfections were carried out using the FuGene 6 transfection reagent according to the manufacture's protocol (Roche, Mannheim, Germany).

Immediately before imaging, chromatin counter stain was obtained by incubating the cells with 1 μg/ml Hoechst 33342 in complete DMEM without Phenol Red for 20 min followed by three times washing with DMEM without Phenol Red. Cells were then kept in complete DMEM supplemented with 20 mM Hepes without Phenol Red. For tracking of nuclear particles we stably transfected SW13 cells with the expression plasmid encoding *Xenopus laevis *GFP-NLS-vimentin. To track vimentin particles in the cytoplasm, SW13 cells were transiently transfected with the *Xenopus laevis *GFP-vimentin cDNA construct described above.

### Mircroinjection of fluorescent polystyrene microspheres

SW13 cells were cultured in P35G-1.5-7-C-Grid cell locate culture dishes (MatTek Corporation, Ashland, MA) in complete DMEM medium for 1 day after plating. Carboxylate-modified 0.1 μm microspheres (FluoSpheres, #F8800, Molecular Probes, Leiden, NL) were obtained as 2 % solids in solution and further diluted to 0.04 % solution in 1 M BSA in PBS. Before microinjection, the microspheres were sonificated for 30 seconds to avoid aggregation. The AIS 2 system (Cell Biology Trading, Hamburg, Germany) was used for microinjection. Injection needles were drawn from borosilicate glass capillaries GC120TF-10 (Harvard Apparatus, Edenbridge, UK) using a Flaming Brown micropipette puller P-97 (Sutter Instruments, Novato, CA). The injection pressure was adjusted to 20–250 kPa in the different experiments. For evaluation of cell viability microinjected cells were grown at 37°C with 5% CO_2 _overnight and examined the next day.

### Imaging

Live cell imaging was carried out on a confocal laser scanning microscope TCS SP2 AOBS (Leica Microsystems, Wetzlar, Germany) using a 63x oil immersion objective with 1.4 optical apperture (HCX PL APO lbd.BL 63x / 1.4, #506192, Leica Microsystems). The microscope was further equipped with a 29 mm objective heater (#0280.010, Leica Microsystems) with temperature – controlled device (#0504.000, Leica Microsystems) and a temperature – controlled fan (ASI 400E, Nevtek, Burnsville, VA, USA). A diode laser (λ = 405 nm) was used for excitation of Hoechst 33342. An argon (λ = 488 nm) and a helium/neon laser (λ = 543 nm) was used for EGFP and fluorescent microsphere excitation, respectively. 3-D image stacks, each consisting of 17 2-D-images, of GFP-vimentin particles and Hoechst 33342 stained chromatin were acquired in parallel at the maximum scanning speed of 1400 Hz (i.e. scan lines per second) with a constant Δt = 10 seconds. Imaging format was set to 256 × 256 pixel, voxel sizes were generally between 0,093 μm × 0,093 μm × 0,325 μm and 0,093 μm × 0,093 μm × 0,450 μm. The laser intensity was adjusted to a minimum to avoid photo damage during imaging. For this purpose the acousto-optical beam splitter (AOBS) were set between 2 – 5% for both lasers with photo multiplier (PMT) settings of 715.7 Volt for the diode laser and 717.4 Volt for the argon and helium/neon lasers. All the image processing steps were carried out in TIKAL (see below).

### Drug treatment

Before drug treatment, we acquired 3-D time series of cells with a time-lapse of Δt = 10 seconds for 10 minutes. Immediately afterwards the medium was exchanged for one of the following solutions: i) 600 mM sorbitol; ii) 20 mM azide / 50 mM deoxyglucose; iii) 0.04 μg/ml nocodazole; and iv) 1 μg/ml cytochalasin D – all in complete DMEM without Phenol Red except for ii), which was applied in PBS [39]. After change of medium, we immediately acquired further 3-D time series images for another 10 min. Thereafter, the drug containing medium was replaced by complete DMEM without Phenol Red. In order to document the recovery and viability of cells, images were acquired for another 10 minutes with the same microscope settings.

### Image registration

4-D image registration [40,41] of consecutively captured three-dimensional images of cell nuclei counterstained with Hoechst 33342 was performed for correcting for global movement of cell nuclei. In order to reduce alignment artifacts due to acquisition noise all three-dimensional image stacks were preprocessed using a 3-D median filter and a 3-D automatic gamma correction for maximum gray value range. Image registration was then performed using an implementation of an automated image registration algorithm [40] running on a high-performance computing cluster. In this study, we only applied rigid and affine transformation since we did not observe drastic local deformations in cellular shape which would require correction by our non-rigid transformation method [23]. Rigid and affine transformation matrices were computed in a two-step process for optimal 4-D object alignment. First, objects at time point *t *+ 1 were consecutively aligned with 3-D objects at time point *t *providing a pre-registered image stack at each time point. Secondly, each pre-registered image stack was aligned with respect to the initial image stack at time point t = 0. Transformation matrices calculated for the chromatin stained images were applied to all corresponding image stacks in the other color channels.

### Tracking beads and vimentin particles

Image processing was carried out using our in-house developed image analysis platform. The analysis chain consisted of three major modules: image preprocessing and segmentation, 4-D tracking and quantification of dynamics, 4-D visualization and user interaction with 4-D data sets. To reduce noise in the vimentin channel, images were subjected to 3-D diffusion filtering [24] followed by segmentation with a pyramid linking algorithm [42]. Particle tracking was performed by extending our already implemented single particle tracking algorithm from 2-D + time to 3-D + time [43,44]. The tracking algorithm uses parameters such as the individual center of masses, volumes, total grey value intensities, velocities and accelerations. To control possible tracking and segmentation artifacts we visualized 4-D tracks of beads and vimentin particles, respectively, together with their isosurface reconstruction at each time point.

### Correlation analysis of chromatin density and tracked nuclear particle

Chromatin images were preprocessed by a 2-D Gaussian smoothing filter to reduce noise. Additionally a gamma filter was applied to use the full grey value range of 8 bits. For additional noise reduction the 256 different grey values were divided into eight grey value classes ranging from 0 – 32, 33 – 65, etc. The centre of mass for each individual polystyrene bead or nuclear vimentin body was calculated and tracked in 3-D over time. For each time point the corresponding binned mean grey value intensities of a 9 × 9 pixel area around the centre of mass was determined.

### Calculation of mean squared displacements and anomalous diffusion coefficients

The mean square displacement (MSD) was calculated for each particle and time point of the data set according to <Δd2> = < [d(t) - d(t + Δt)] 2> and plotted as <*d*^2^> (μm^2^) versus Δ*t *(s) using Matlab (The MathWorks, Inc., Novi, MI). Further evaluation of anomalous diffusion (α) [25,26] was determined by using the regression curve fitting functions implemented in Matlab.

### TIKAL image processing platform

TIKAL is an in-house developed platform for multi – purpose image processing (executable code can be requested at: ). The program consists of a graphical user interface for easy access of the underlying algorithms. The software is written in C/C++ and can be deployed both on Linux and Windows systems. The main components of the program are separated into several main modules, namely a filter, registration, tracking, visualization and data handling module. The filter module contains 2-D and 3-D image processing algorithms such as gamma correction, median, gaussian, anisotropic diffusion filters [24]. Segmentation filters range from simple threshold operations to complex pyramid linking segmentation [42]. The registration module is capable of handling single 3-D image stacks as well as 4-D time-lapse series of image stacks. Image stacks can be corrected by a combination of rigid and affine transformations as well as non-linear correction of local deformations by registration based on a thin plate spline model [22,23]. The tracking [44] and visualization part can be combined to allow the user a visual inspection of the tracking result and enable a correction of falsely assigned trajectories. The tracking and visualization module contains functions for calculation of quantitative parameters such as distances, velocities and mean squared displacement (MSD).

The software is capable to import both the proprietary Leica and the commonly used Tiff file format. Currently we are working on an implementation to integrate and combine our software with the Open Microscopy Environment, OME [15]. This is of particular importance when working with huge data sets, when either computer memory or disk space become limiting factors in the analysis stream.

## List of abbreviations

### Expression plasmids and construction

NLS – Nuclear localization sequence

GFP – green fluorescent protein

## Authors' contributions

CPB wrote the program for 4-D data analysis (TIKAL). With this platform he was able to perform data analysis and data evaluation. Further, microinjection of cells and nuclei and their respective imaging was carried out by CPB. In additions he assisted MR with imaging and cell culture of the vimentin transfected SW13 cells.

MR performed the transfection, culturing, imaging and inhibitor treatment of SW13 cells. Additionally she contributed to the interactive 4-D tracking analysis. CA was involved in planning of this study at an earlier stage. HH supervised the experimental part, whereas RE was responsible for the entire setup and planning of the computational part of this study.
